# Root Pulling Force Across Drought in Maize Reveals Genotype by Environment Interactions and Candidate Genes

**DOI:** 10.3389/fpls.2022.883209

**Published:** 2022-04-15

**Authors:** Patrick Woods, Kevin R. Lehner, Kirsten Hein, Jack L. Mullen, John K. McKay

**Affiliations:** ^1^Department of Agricultural Biology, Colorado State University, Fort Collins, CO, United States; ^2^Graduate Degree Program in Ecology, Colorado State University, Fort Collins, CO, United States

**Keywords:** maize (*Zea mays* L.), root systems, candidate genes, phenotypic plasticity, drought stress, genome wide association studies (GWAS)

## Abstract

High-throughput, field-based characterization of root systems for hundreds of genotypes in thousands of plots is necessary for breeding and identifying loci underlying variation in root traits and their plasticity. We designed a large-scale sampling of root pulling force, the vertical force required to extract the root system from the soil, in a maize diversity panel under differing irrigation levels for two growing seasons. We then characterized the root system architecture of the extracted root crowns. We found consistent patterns of phenotypic plasticity for root pulling force for a subset of genotypes under differential irrigation, suggesting that root plasticity is predictable. Using genome-wide association analysis, we identified 54 SNPs as statistically significant for six independent root pulling force measurements across two irrigation levels and four developmental timepoints. For every significant GWAS SNP for any trait in any treatment and timepoint we conducted *post hoc* tests for genotype-by-environment interaction, using a mixed model ANOVA. We found that 8 of the 54 SNPs showed significant GxE. Candidate genes underlying variation in root pulling force included those involved in nutrient transport. Although they are often treated separately, variation in the ability of plant roots to sense and respond to variation in environmental resources including water and nutrients may be linked by the genes and pathways underlying this variation. While functional validation of the identified genes is needed, our results expand the current knowledge of root phenotypic plasticity at the whole plant and gene levels, and further elucidate the complex genetic architecture of maize root systems.

## Introduction

Originating approximately 400 million years ago, roots evolved at least twice in early plant lineages and provided anchorage (Raven and Edwards, 2001). Following these origins, roots continued to evolve structural complexity and functions and serve many critical roles in the biology of plants. In addition to water and nutrient uptake, roots are responsible for anchorage in soil. Roots are also the site of rhizosphere biotic interactions, spanning a range of outcomes from pathogenic to beneficial, and involving microbes, insects, and other plants (Johnson and Rasmann, 2015; Lareen et al., 2016; Clarke et al., 2019). Despite these important roles, understanding the genetics and physiology of root systems in soils has been challenging, leading to the description of roots as the “Hidden Half” of the plant (Eshel and Beeckman, 2013). Overall, root systems have complex structures. Root System Architecture (RSA) is a term used to describe the spatial arrangement of multiple individual roots of several distinct root types of an individual plant, each at a different stage along its developmental trajectory (Smith and de Smet, 2012). RSA traits, then, are the result of the cumulative effects of development of many individual roots within an individual of a single genotype in a given environment.

In addition to their fundamental biological importance and intrinsic structural complexity, roots and root systems vary among species, genotypes, and environments. Within a given species, RSA is highly plastic, responding to variation in nutrient status and soil composition (Karlova et al., 2021). RSA traits have been shown to respond to levels of nutrients such as phosphorus and nitrogen by regulating root growth and branching (Gaudin et al., 2011; de Jong et al., 2019). Differences in particle texture within a growth substrate have also been shown to affect RSA phenotypes (Rogers et al., 2016). Water availability regulates both timing of root development and directional patterns of root branching (Bao et al., 2014; Sebastian et al., 2016). These examples highlight our growing understanding of the robust and diverse responses of root growth to environmental stimuli.

Phenotypic plasticity in the form of altered rates of growth and timing of development of organs and traits are common adaptive responses for both RSA and above ground traits in plants (White and Castillo, 1992; Strock et al., 2018). While phenotypic plasticity is a property of an individual, the degree to which individuals sense and respond to the environment can be represented as a genetic component (Falconer, 1990). This variation in how genotypes sense and respond to the environment is described statistically in an ANOVA as a genotype by environment interaction (GxE). GxE is a common property of quantitative traits (Lynch and Walsh, 1998), affecting both the range of phenotypic values and the rank of genotypes in different environments. Multi-environment genetic mapping studies have shown that this GxE is polygenic and can be resolved to individual genome regions and loci, where the effect size of quantitative trait loci (QTL) changes across different environments (Paterson et al., 2003; El-Soda et al., 2014; Lowry et al., 2019).

Phenotypic plasticity for agriculturally important traits, has been proposed as a breeding target for optimizing response to environmental stress (Nicotra and Davidson, 2010; Kusmec et al., 2017; Kusmec et al., 2018). Implementing this strategy will require a deeper understanding of the extent and the genetic underpinning of GxE for the key traits. The core function of roots in obtaining water and nutrients highlights the potential for utilizing RSA in crops to buffer against environmental volatility due to climate change (Voss-Fels et al., 2018). Root phenotypic plasticity has an unclear, and potentially complicating, role in RSA under variable water and nutrient conditions (Schneider and Lynch, 2020). Future breeding efforts for RSA will be aided by a fuller understanding of GxE interactions affecting roots.

Like many traits, our understanding of the fundamental genetic control of RSA has largely been driven by studies in the model dicot *Arabidopsis thaliana* (Petricka et al., 2012). In addition to elucidating the core conserved pathway of root development and signaling, genetic loci associated with GxE responses of Arabidopsis roots to nutrient stress have been identified (Rosas et al., 2013). In contrast with the relatively simple roots of Arabidopsis, root systems of important monocot cereal crops such as maize, wheat, and rice have fundamentally different and more complex structures (Smith and de Smet, 2012). Accordingly, our understanding of the genetic architecture of RSA in these crop species is poor in comparison to Arabidopsis and related dicots. Most studies examining the genetics of RSA in cereal crops have been done on young plants grown in controlled conditions (Tracy et al., 2020).

Relatively few studies have attempted to characterize the genetic architecture and phenotypic plasticity of RSA in mature field grown cereal crops due to the challenges of measuring these traits. Destructive phenotyping of roots using excavation followed by image-based techniques have been effective in mapping RSA traits across different crop species (Trachsel et al., 2011; Schneider et al., 2020; Zheng et al., 2020). Analysis tools to extract phenotypic data from images of excavated root systems have been developed (Das et al., 2015; Zheng et al., 2020). Loci associated with variation in maize RSA traits have been identified in two recent genome wide association studies (GWAS), both of which used shovelomics (Trachsel et al., 2011) based techniques for root system excavation (Schneider et al., 2020; Zheng et al., 2020). Schneider et al. (2020) used two different diversity panels across 3 years in two locations including two levels of irrigation at one of these locations and measured image-based RSA traits such as lateral root length and root angle. Their multi-year and multi environment phenotyping efforts were followed by GWAS analysis in FARMCPU which identified candidate genes associated with variation in both the plasticity and mean trait values within each environment. Zheng et al. (2020) used the shoot apical meristem (SAM) diversity panel in a single environment and growing season. While their efforts did identify candidate genes associated with numerous image-based RSA traits, the lack of multiple environments and growing seasons in Zheng et al. (2020) motivates further use of the SAM population to investigate the plasticity and genetic control of RSA. Overall, the consensus from these shovelomics based GWAS studies is that maize RSA is highly polygenic, controlled by multiple low to moderate effect loci, and that GxE is a strong component of RSA genetics because of the inconsistent set of identified candidate genes.

We focus on root pulling force (RPF) which is an alternative and a higher throughput technique compared to shovelomics based excavation for removing root systems from a field. RPF is quantified through the process of attaching the base of the shoot with a rope to a digital force gauge and applying manual, vertical force until the root system is extracted from the soil. The force required to pull the root system out of the ground is measured by the force gauge and recorded as RPF. This method provides a simple, instantaneous and quantitative measurement of the roots system during extraction. We have successfully used the RPF technique to identify QTL for root system size in *Brassica napus* (Fletcher et al., 2015), and QTL for it have been found in maize (Lebreton et al., 1995). While RPF can serve as a simple measure for root architecture traits, the extracted root systems remain amenable to image-based measurements as in excavation-based root extraction. Imaging of pulled root systems has shown RPF to be highly indicative of root system volume and surface area (Shao et al., 2021). Due to the simplicity and individual plant sampling of the RPF method, it is also amenable to automation to increase the throughput of RSA measurements in the field for improved mapping studies.

Here we used RPF, along with subsequent imaging of pulled root systems, to examine means and plasticity of RSA traits in the SAM diversity maize panel. We phenotyped this population at multiple developmental stages across two field seasons and differential irrigation treatments to investigate GxE in RSA traits. We then performed GWAS on these measurements, which identified a number of candidate genes potentially underlying the variation in root traits. Important questions addressed in this work are: (1) What proportion of trait variance in RPF and RSA in maize is explained by genetics, the environment and GxE? (2) What is the genetic architecture of RPF and RSA in maize across development and irrigation treatments? (3) Is there evidence for GxE at the individual gene level?

## Materials and Methods

### Field Experiments

**FIGURE 1:**
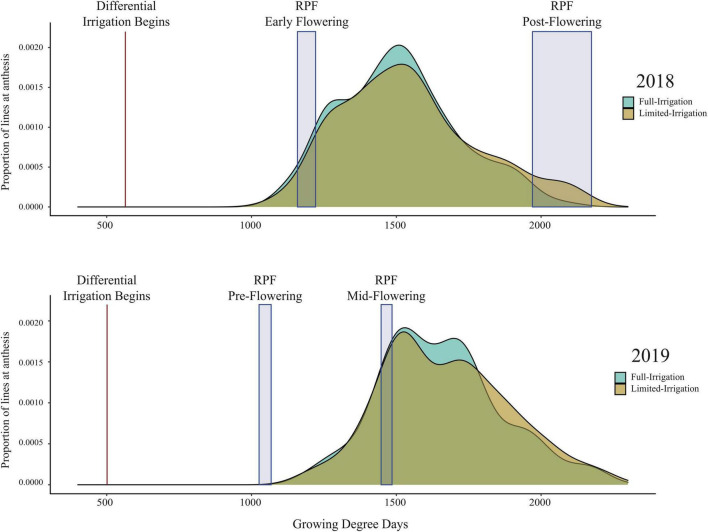
Histogram of days to anthesis for the field experiments. The timing of the root pulling events are highlighted and assigned developmental stages relative to flowering: pre-flowering for the pulling at 71–73 days after planting, prior to any lines reaching anthesis, early flowering at 61–66 days after planting when roughly 5% of lines had reached anthesis, mid-flowering at 91–93 days from planting when roughly 30% had reached anthesis, and post-flowering at 109–119 days from planting when 98% of lines had reached anthesis.

In a survey, three hundred and sixty-seven lines from the SAM diversity panel (Leiboff et al., 2015) and 4 inbred check lines were grown at the Colorado State University Agricultural Research Development and Education Center in Fort Collins, CO, United States (40.649 N, −105.000 W) in 2018 and 2019. In 2018 seeds were planted on 22–25 May and in 2019 on 14 May using a split-plot design with full irrigation (FI) or limited irrigation (LI) treatments, with three field replicates per treatment. Prior to planting, the fields were fertilized to recommendations for 200 bu/ac yield, amounting to 190 lb/ac N and 25 lb/ac P_2_O_5_ in 2018 and 190 lb/ac N, 60 lb/ac P_2_O_5_, and 4 lb/ac Zn in 2019. Each plot consisted of two 12-foot rows with 30-inch spacing between rows and 9-inch spacing between plants within rows. The irrigated treatments received approximately 1 inch of water per week, while the drought treatments were irrigated until well-established (~5 weeks after planting) and then received only natural precipitation, except at the root harvesting when it also received irrigation to homogenize the root harvesting process (Supplementary Table 1). This irrigation differential began 500–600 growing degree days after planting, a time in which the average developmental stage was approximately V4. The timing of the root harvests was categorized developmentally relative to the timing of anthesis in the population (Figure 1), pre-flowering for the pulling at 71–73 days after planting, prior to any lines reaching anthesis, early flowering at 61–66 days after planting when roughly 5% of lines had reached anthesis, mid-flowering at 91–93 days from planting when roughly 30% had reached anthesis, and post-flowering at 109–119 days from planting when 98% of lines had reached anthesis. The variation in RPF timepoints allowed us to assess how the various developmental stages might affect root system excavation in this diverse population. This information will improve the efficiency of future root-pulling events. Days to anthesis was measured for each plot as the date at which 50% of the plants in the plot had begun to shed pollen and was determined approximately 3 times per week.

For root phenotyping, a rope was attached to the stalk above the root crown with a slip knot, and a digital force gauge (DS2, Imada Inc., Northbrook, IL, United States) was used to pull the root crown vertically from the soil. Root crowns were cleaned with water and imaged to obtain additional RSA measurements using the DIRT image analysis software (Das et al., 2015) before being air-dried to constant weight for measurement of mass.

### Genetic Correlations

Spearman’s rank correlation coefficients were calculated using the ‘‘chart.Correlation’’ function in the ‘‘Performance Analytics’’ package in R.^1^

### Quantitative Genetic Analyses

We estimated broad-sense heritability of traits using a random effect one-way ANOVA model. We split phenotypic data by trait, timepoint, and treatment and used the lmer function in the R package “lme4” v1.1-21 (Bates et al., 2015), treating genotype as a random effect. Additionally, we estimated phenotypic variance due to genotype, the environment and GxE by using a random effect two-way ANOVA model with interaction using the lmer function from the R package “lme4” v1.1-21. Genotype, the environment and GxE were all treated as random effects in the model.

### Genome Wide Association Analysis

To analyze phenotype data *via* GWAS, the best linear unbiased predictors (BLUPs) were calculated from genotype simple means by treating genotype as a random effect using the R package “lme4” v1.1-21 (Bates et al., 2015) (see Supplementary Data Sheet 1 with BLUP values used for GWAS). A genotype matrix in HapMap format containing 1.2 million single nucleotide polymorphism (SNP) calls for the SAM diversity panel was then obtained from Leiboff et al. (2015). To reduce the impact of rare erroneous SNP calls, we imposed a minor allele frequency filter of 5% on the genotype matrix. After filtering for minor allele frequency, the genotype matrix contained approximately 860,000 SNPs. GWAS was conducted using an R implementation of FarmCPU (Liu et al., 2016) *via* GAPIT (Lipka et al., 2012) by following the guidelines at https://www.zzlab.net/GAPIT/. The first three principal components as well as a kinship matrix calculated using GAPIT were used as covariates to control for population structure. Normality of p-value distributions were assessed by inspecting each trait’s Q-Q plot output by GAPIT (Supplementary Figure 1). Because each trait’s Q-Q plot displayed p-value distributions that followed approximate normality, we proceeded with the FarmCPU results. SNPs whose significance passed the Benjamini—Hochberg false discovery rate threshold (0.05) were further investigated to identify the gene or genes within a 10-kb window with which they may be associated.

### Statistical Tests for Significant Genome Wide Association Studies Hits

To identify QTLxTreatment effects among significant GWAS hits, we performed ANOVA on each of these SNPs. We constructed models using the lm function in R. We estimated the effects of SNP, treatment, and SNP-by-treatment interactions. We performed PCA analysis using our SNP genotype data. We included values from the first three principal components in each ANOVA to account for population structure among genotypes. Using the Anova() function from the R package “Car” (Fox and Weisberg, 2018) we performed a type 3 ANOVA.

Gene expression data are from Sekhon et al. (2011). We compared mean normalized expression for root tissue and pooled leaves from the V1-stage plants.

## Results

### Root Pulling Force Measures Root System Size and Is Correlated With Root System Architecture Traits in the Field Across Multiple Developmental Timepoints

**FIGURE 2:**
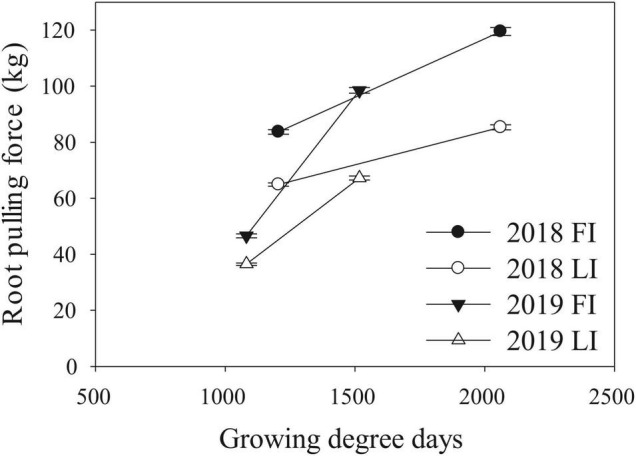
Changes in RPF over the field growing seasons. Measurements for the two field seasons are given (mean ± SE) for the full (FI) and limited (LI) irrigation treatments.

**FIGURE 3:**
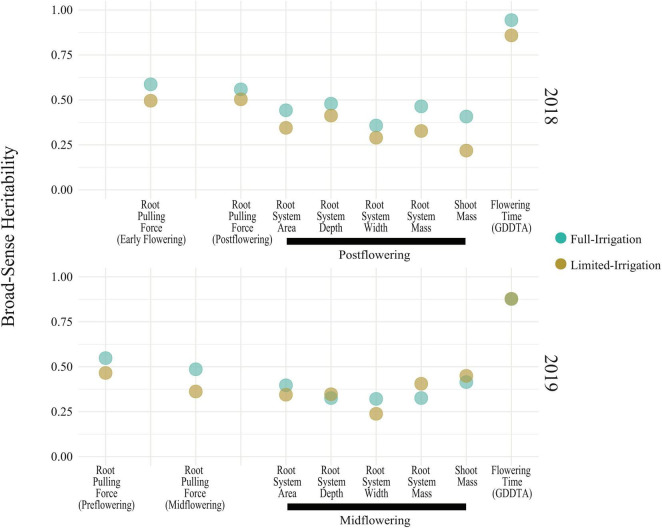
Broad-sense heritability of RPF and other root traits in the field.

We performed a field experiment during Summer 2018 and 2019 in Fort Collins, Colorado using 367 diverse lines from the SAM maize panel (Leiboff et al., 2015) with full irrigation (FI) and limited irrigation (LI) treatments. We measured RPF at four different timepoints across 2018 and 2019, roughly corresponding to pre-flowering, early flowering, mid-flowering, and post-flowering (see Section “Materials and Methods”). RPF measurements for the SAM population predictably increased with time with a mean of 47 kg pre-flowering to a mean of 120 kg at the end of season, slowing after flowering (Figure 2). Plants in the LI treatment had lower RPF measurements, averaging 70–80% of the full-irrigation (FI) RPF values. Above-ground biomass was also reduced in LI, 60–90% of FI across the four stages. We estimated the broad-sense heritability of RPF as H2 = 0.49–0.59 in the FI treatment, and generally lower estimates for the LI treatment (H^2^ = 0.36–0.50, Figure 3), but sufficient for mapping experiments.

**FIGURE 4:**
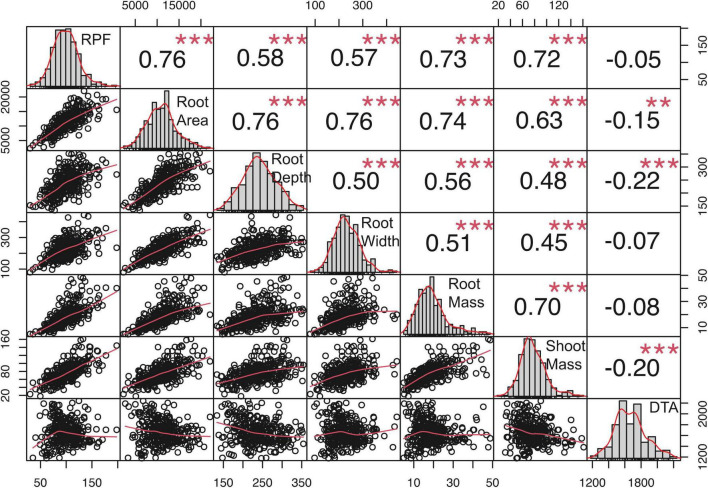
Genotypic correlations among traits in the 2019 full-irrigation treatment, mid-flowering. Values are Spearman’s correlation coefficients. ** and *** indicate significant correlations, *P* < 0.01 and 0.001, respectively.

To quantify covariance between RPF measurements and RSA traits, We conducted Spearman’s correlation analysis, which showed that RPF measurements were highly positively correlated with RSA traits at all timepoints and treatments (Figure 4 and Supplementary Tables 2, 3). RPF showed the greatest positive correlation with root mass and area. This is consistent with our previous study of 3D X-ray tomography measurements from the root crowns of a subset of SAM lines, where RPF was highly associated with root volume and surface area (Shao et al., 2021). As further evidence for the utility of RPF as a sampling method, estimates of heritability for RPF were greater than or equal to those for RSA traits (Figure 3). In addition to root traits, we measured the shoot mass of pulled plants and recorded flowering time as growing degree days to anthesis (GDDTA) of the SAM panel. Shoot mass was highly positively correlated with RPF and other RSA traits (Figure 4 and Supplementary Tables 2, 3). Flowering time has a more variable and overall weaker correlation with RSA and plant biomass traits across timepoints and treatments (Supplementary Tables 2, 3).

To assess the relative utility of the root systems that we extracted by RPF, we extracted root systems from a subset of 45 plots using both RPF and shovel-based excavation methods (Trachsel et al., 2011). The mass of root systems extracted from the two techniques was similar (Supplementary Figure 2A), and genotypes varying in RSA showed consistent differences in plant form with both techniques (Supplementary Figure 2B).

### There Is a Wide Range of Plasticity in Root Pulling Force in Response to Water Limitation Across the Shoot Apical Meristem Panel

**FIGURE 5:**
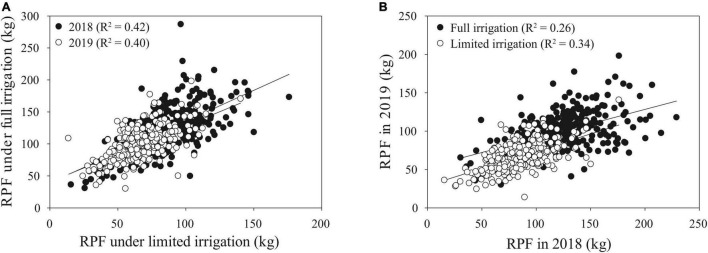
Genotypic correlation for RPF of lines among environments. **(A)** Correlations between treatments, for 2018 *R*^2^ = 0.42 and for 2019 *R*^2^ = 0.40. **(B)** Correlations between years, for FI *R*^2^ = 0.26 and for LI *R*^2^ = 0.34. Data shown are from the post-flowering stage in 2018 and mid-flowering in 2019.

**FIGURE 6:**
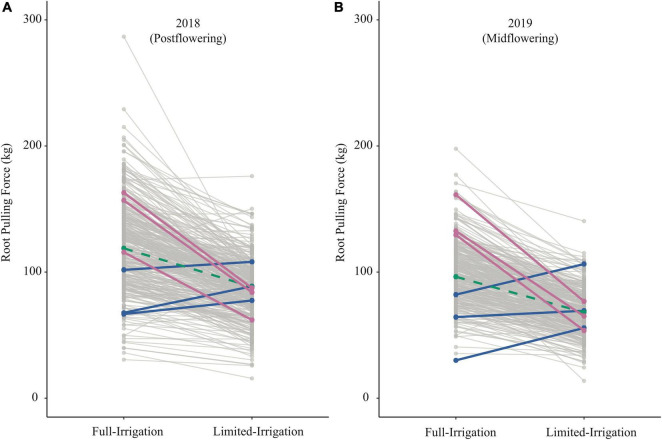
Reaction norms for RPF across irrigation treatments in 2018 **(A)** and 2019 **(B)**. Blue lines highlight three genotypes (I205, H91, and S8324) that consistently increased RPF under LI; Pink shows three genotypes (A661, 207, and PHN11) where RPF decreased strongly in both years under LI; Green indicates genotype 793, which was approximately average for response to LI.

For RPF, we found a moderate genotypic correlation between irrigation treatments and field seasons (Figure 5), suggesting that there may be differences among genotypes in the SAM population for plasticity to water limitation. For RPF, there was a high average plasticity response across the population, with an overall reduction in RPF under LI treatments (Figure 6). Among individual genotypes, however, there was a large range of plasticity of RPF in response to water limitation. As examples, we highlight six genotypes with consistent responses to irrigation in RPF across field seasons (Figure 6). These groups of genotypes showed large differences in plasticity. One group exhibited a large reduction in root system size under limited irrigation, while the other had increased root size under water limited conditions.

Across treatments and growing seasons, variance component analysis indicated that GxE accounted for a range of 1.2–9.8% of the phenotypic variation in RPF (Supplementary Figure 3). Overall, across years we found a significant correlation in plasticity in RPF to water limitation, on a percent of wet treatment basis (*r* = 0.2, *P* < 0.01). This plasticity in RPF is positively associated with plasticity in shoot mass, even on a percent of wet treatment basis (*r* = 0.4–0.6, *P* < 0.001).

### Genome Wide Association Analysis Identifies Numerous Single Nucleotide Polymorphisms Associated With Root Pulling Force and Root System Architecture Traits

The FarmCPU GWAS model controls for false discoveries by accounting for kinship and population structure while still providing high statistical power for candidate gene identification (Liu et al., 2016). Additionally, the relatively rapid LD decay in maize results in good resolution in mapping through GWAS (Yan et al., 2009). We performed GWAS using an R implementation of FarmCPU through GAPIT (Lipka et al., 2012) to identify SNPs associated with RPF, RSA, biomass traits, and flowering time. We identified 54 significant GWAS SNPs for RPF across years, developmental timepoints, and treatments, along with 6 for root mass, 3 for shoot mass, and 21 for RSA traits (Supplementary Table 4). For RPF, the significant GWAS SNPs varied between treatments with more SNPs identified in FI than LI. None of the RPF significant GWAS SNPs overlapped between the treatments, which may be due to GxE, but is also expected given the way that FarmCPU selects SNPs to include in the model. In addition, we also found 48 significant GWAS SNPs for days to anthesis (Supplementary Table 5); however, we saw no overlap in hits between our root traits and flowering, consistent with the lack of correlation in Figure 4.

The quantitative genetic analysis of RPF revealed GxE for drought (Supplementary Figure 3). In addition, we saw lack of overlap of QTL between FI and LI treatments within a timepoint for RPF and other root traits. This is in contrast to flowering time where GxE effects appear minimal (Supplementary Figure 3) and GWAS analysis identified hits in common between FI and LI treatments (Supplementary Table 5). To test for QTLxTreatment effects for the quantitative traits we measured, we performed a *post hoc* ANOVA on each of the 54 significant GWAS SNPs for RPF that we identified. We tested effects of SNP, treatment, and SNP-by-treatment interaction. To account for potentially confounding effects of population structure, we included PCA scores generated from SNP data in the ANOVA model. Through this analysis, we found that eight of the significant GWAS SNPs for RPF showed a significant QTLxTreatment effect (Supplementary Table 4). In contrast, none of the GWAS SNPs for flowering time had a significant QTLxTreatment effect (Supplementary Table 5).

**FIGURE 7:**
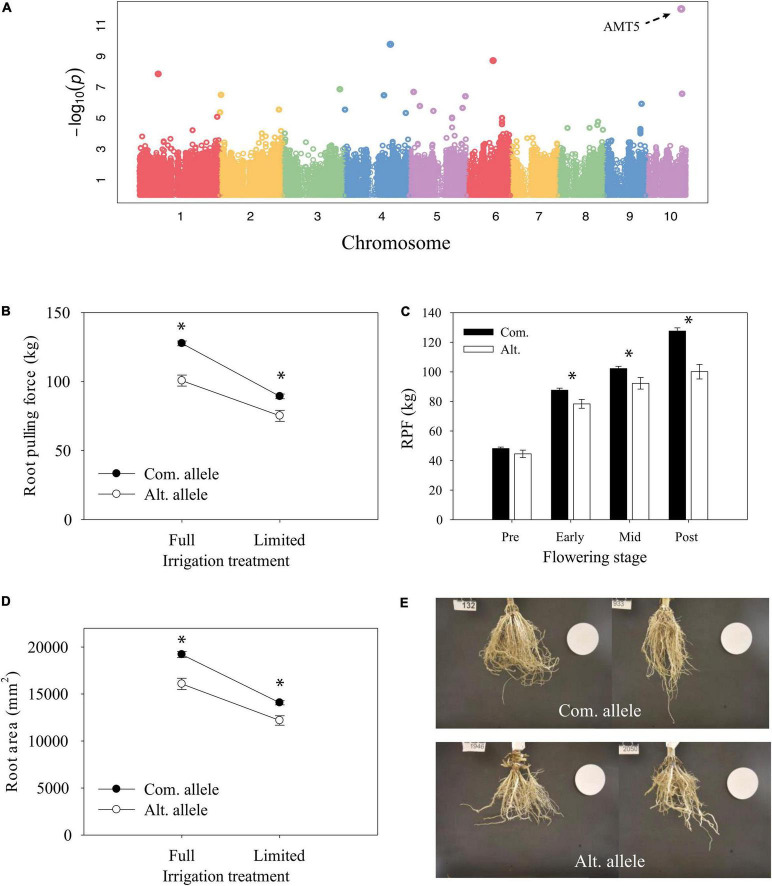
Characterization of candidate gene *AMT5*. **(A)** Genome-wide association mapping of RPF under well-irrigated conditions at 2018 post-flowering stage. **(B)** Reaction norm of RPF from 2018 post-flowering measurements for lines with contrasting alleles (ls mean ± SE). **(C)** Differences in RPF by allele across developmental time points for the full-irrigation treatment (mean ± SE). * Indicates a significant difference, *P* < 0.05. **(D)** Reaction norm of root area (mean ± SE). **(E)** Representative root images for lines with the common allele (top) or alternate allele (bottom).

**FIGURE 8:**
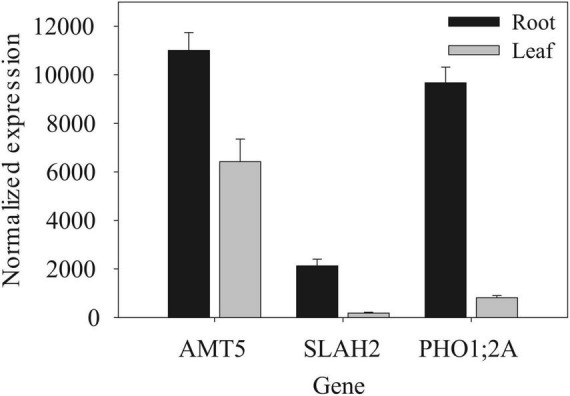
Gene expression in root and shoot organs among nutrient transport candidate genes. Data (mean ± SD) are for V1-stage plants from Sekhon et al. (2011).

The most significant GWAS hit for root traits was on chromosome 10 and identified from GWAS of the full-irrigation RPF measurement in 2018 (Figure 7A). The candidate gene Zm00001eb427000 is the only gene model within a 10-kb window of the significant GWAS SNP and characterized as the low-affinity ammonium transporter *AMT5* (Dechorgnat et al., 2019) which shows a prominent root specific expression profile (Figure 8). The alternate allele was associated with lower RPF not only in the FI treatment but also in the LI treatment (Figure 7B). However, the effect size of this polymorphism on RPF was smaller in the dry treatment, with significant GxE at this trait associated SNP. Although this SNP was only reported significant in FarmCPU at a single developmental stage, we found that allele-specific differences in RPF began to emerge at early flowering, but it may be that the effect sizes of this polymorphism varies across development (Figure 7C). The differences in RPF that we observed captured differences in other RSA traits such as root area and depth (Figures 7D,E).

**FIGURE 9:**
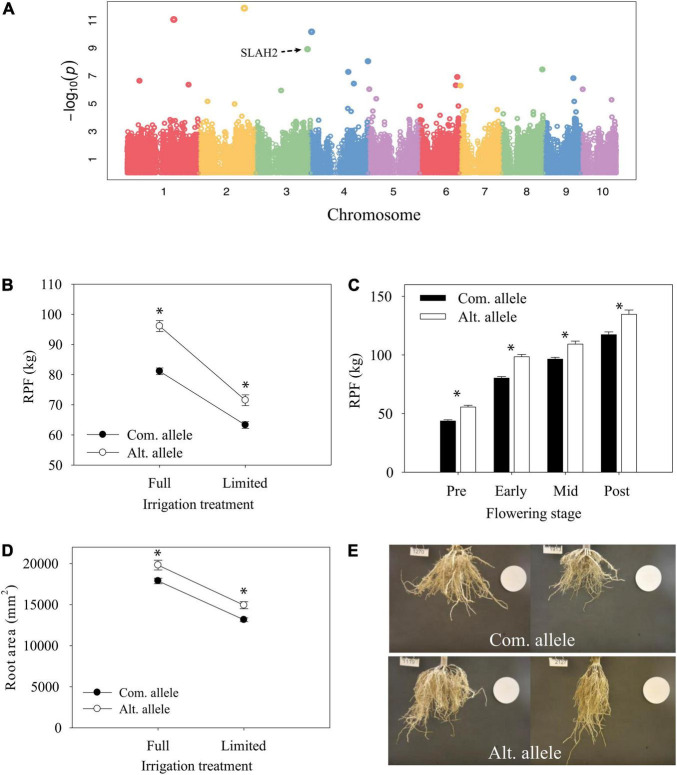
Allele differences at candidate gene *SLAH2*. **(A)** Genome-wide association mapping of RPF under well-irrigated conditions at 2018 early-flowering stage. **(B)** Reaction norm of RPF from 2018 early-flowering measurements for lines with contrasting alleles (ls mean ± SE). **(C)** Differences in RPF by allele across developmental time points for the full-irrigation treatment (mean ± SE). * Indicates a significant difference, *P* < 0.05. **(D)** Reaction norm of root area (mean ± SE). **(E)** Representative root images for lines with the common allele (top) or alternate allele (bottom).

Another significant GWAS SNP showing significant GxE during early flowering was within an exon of the gene Zm00001eb159490, a *SLAH2* nitrate channel (Figures 9A,B). Although the significant GWAS SNP was found at the early-flowering stage, the alternate allele was associated with significantly higher (*p* < 0.05) RPF at all developmental stages sampled (Figure 9C). Image analysis of root systems indicated that there were significant differences in root system depth and area between the alleles at the significant GWAS SNP (Figures 9D,E). *SLAH2* also has a prominent root specific expression profile (Figure 8), in line with the root phenotypes we observe. This is consistent with localization in Arabidopsis where the homologs are found predominantly in the root stele (Zheng et al., 2015).

**FIGURE 10:**
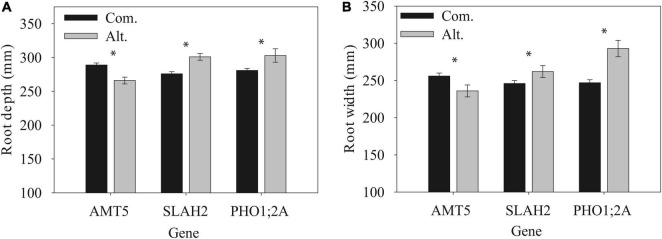
RSA traits of nutrient channel candidate genes, root system depth **(A)** and width **(B)**. Data are mean ± SE for the common (Com) and alternate (Alt) alleles under full irrigation post-flowering. * Indicates a significant difference between alleles, *P* < 0.05.

Another notable candidate genes for RPF was *phosphate transporter 1;2A*, *PHO1;2A* (Supplementary Table 4), is also a nutrient carrier. It shows a similar root specificity with the two root candidates mentioned above (Figure 8). Similar to *SLAH2*, *PHO1;2A* shares a similar localization to the root stele (Hamburger et al., 2002). Additionally, like *AMT5* and *SLAH2*, the significant GWAS SNP in *PHO1;2A* was also associated with differences in basic RSA traits such as root system depth and width (Figure 10). The allele effects in *PHO1;2A* had a significant effect (*p* < 0.05) on root system width. This is consistent with the role of *PHO1;2A* in phosphorus transport, given phosphorus signaling’s role in regulating shallow root angles (Liao et al., 2001). These candidates highlight the important role of nutrient signaling in the shaping of RSA.

## Discussion

### Trait Correlations and Plasticity of Maize Root System Architecture

Correlated phenotypes often indicate the presence of co-located QTL with pleiotropic effects (Otto, 2004). While we did identify numerous significant correlations between RSA phenotypes, only the irrigated post-flowering RPF and root area traits shared a candidate gene (*flz35*, Zm00001eb428170). The consistent lack of co-located candidate genes across all the RSA traits suggests that despite being correlated, independent phenotypic variation was present which allowed our analyses to identify candidate genes uniquely associated with these trait’s measurable variation. We note however that GWAS models such as FarmCPU are not optimal for explicitly testing whether the same loci are affecting traits across developmental stages, environments or growing seasons and thus may be one reason our GWA efforts did not identify overlap in RSA candidate genes. Schneider et al. (2020) and Zheng et al. (2020), who also used FarmCPU for their GWAS analyses, identified a similar phenomenon of few co-localization of root phenotype candidate genes. The lack of shared candidate genes across years could also be due to differences in the developmental stages at which the plants were phenotyped, and we did observe differences in allelic effect sizes at different developmental stages (Figures 7, 9C). Despite this, our analyses focused on identifying GxE across irrigation treatments that could be affecting our candidate gene list. Thus, we implemented *post hoc* tests for GxE for all significant GWAS SNPs and identified 12 SNPs that showed significant GxE: 8 for RPF, 2 for shoot mass, and 1 each for root mass and area. Although we identified 48 candidate genes for flowering time, none showed significant GxE which is reflective of this trait’s overall lower phenotypic plasticity (Supplementary Figure 3).

Understanding the genetic basis of how crops produce alternative phenotypes in response to heterogenous environments can provide an immense source for crop improvement through breeding (Kusmec et al., 2018). Through our analyses, we found extensive evidence for root phenotypic plasticity among maize lines across growing seasons. While the majority of genotypes (>50%) exhibited lower RPF under drought conditions, a subset of genotypes displayed consistent increases or decreases in their RPF from irrigated to drought conditions across growing seasons (Figure 6). The consistent pattern of RPF plasticity we find suggests that root plasticity is predictable for some maize genotypes across growing seasons and thus heritable, as also found by Schneider et al. (2020). This finding lends credence to the notion that maize breeding programs can breed for genetics that reliably enhance the adaptive value of RSA to a specific target population of environments.

Additionally, our analyses were also able to identify more fine-scale evidence for phenotypic plasticity at the individual gene level (Figures 7, 9 and Supplementary Table 4). Interestingly, of the four candidate genes for RPF exhibiting significant GxE that also had functional annotation, two were genes involved in nitrogen signaling (*AMT5*, Zm00001eb427000 and *SLAH2*, Zm00001eb159490). Our finding of nitrogen signaling genes exhibiting GxE in response to drought is supportive of previous reports showing differential expression of homologous nitrogen signaling genes in response to water stress in other plant species (Bielsa et al., 2018; Araus et al., 2020; Filiz and Akbudak, 2020). We hypothesize that the alternate SNP alleles in these nitrogen signaling genes may be linked to additional polymorphisms that differentially affect transcriptional responses of these genes to water availability which underlie their gene level GxE. Furthermore, the evidence for GxE we identified could indicate that these candidate genes are involved in regulating a synergistic response to nitrogen and water availability, which can affect the capture of both as suggested by Araus et al. (2020).

### Candidate Genes Underlying Maize Root Pulling Force Variation

Two recent field-based studies quantified variation, covariation and the genetic architecture underlying variation in maize RSA using GWAS (Schneider et al., 2020; Zheng et al., 2020). Schneider et al. (2020) utilized a diversity panel consisting of different genotypes coupled with differential irrigation treatments at different environments. Their results generate a number of mechanistic hypotheses on the adaptive value of specific root traits in particular target environments. For example, they hypothesize that phenotypic plasticity for root angle is potentially more advantageous for plants in environments that experience prolonged stress such as nitrogen deprivation. Schneider et al. (2020) also present a GWAS analyses which highlighted auxin related genes as being responsible for phenotypic variation in resource rich environments. They also found candidate genes related to cytokinin and phosphorus metabolism that were associated with variation in root plasticity that differed from loci controlling variation in a given environment. Zheng et al. (2020) performed their analyses using the same genotypes used in our study but in a single environment and growing season. They also presented GWAS analysis in FarmCPU that identified a candidate gene encoding a MATE transporter with a known function in shaping maize RSA. Key differences between these prior studies and ours include the testing environments, developmental stages, and method of acquiring root systems (shovelomics vs. RPF). Our RPF sampling study was performed in two growing seasons in Colorado which is located in the high plains region of the U.S. which frequently experiences drought and thus serves as an ideal location to study genetics related to drought stress in crops. Additionally, using RPF to acquire root systems as opposed to shovelomics techniques (Trachsel et al., 2011) is advantageous because this method is less prone to missing data and provides an additional quantitative measure of RSA that is generally more heritable than image-based root traits (Figure 3) and has been used previously to identify QTL for drought responses in maize (Lebreton et al., 1995).

Our GWAS efforts identified a total of 54 SNPs associated with measurable variation in RPF across developmental stages, irrigation treatments, and growing seasons (Supplementary Table 4). Most of these 54 SNPs were located within gene models whose annotation spanned a diversity of functions such as monosaccharide transport (Zm00001eb166700), insect resistance (Zm00001eb273440), and auxin biosynthesis (Zm00001eb060250). Interestingly, our GWA efforts found no overlap of candidate genes across developmental stages, irrigation treatments, or growing seasons for RPF despite their similar allelic effects identified from *post hoc* analyses (Figures 7C, 9C). The lack of overlap and quantity of functionally diverse RPF candidate genes we find suggest a highly complex genetic architecture for this trait that is determined by numerous factors including developmental stage, environment, and growing season. We hypothesize that the pathway controlling root development may be conserved, but genotypes may vary in pathways that provide environmental signals and at different times of development to the core development pathway. Future studies should design experiments to mechanistically understand when and why these candidate genes affect RPF dynamics at specific developmental stages and environments.

Our GWAS analysis identified candidate genes related by their: (1) roles in nutrient signaling from roots to shoots and (2) greater expression in root tissue such as *AMT5*, *SLAH2*, and *PHO1;2A*, Zm00001eb191650 (Maierhofer et al., 2014; Salazar-Vidal et al., 2016; Filiz and Akbudak, 2020). This is interesting because despite our experiment being conducted in a field that was fully fertilized, SNPs in genes involved in the transfer of the supplemented nutrients were significantly associated with measurable variation in RPF. As previously indicated by Schneider et al. (2020), this phenomenon suggests the presence of allelic variation in these candidate genes that significantly affects their ability to respond to soil nutrients. We hypothesize that these candidate genes possess alleles that result in alternate signals of nutrient starvation/supplementation which causes root systems to grow differentially in response—underlying the heritable variation we observed in RPF. Previous studies have shown that in *A. thaliana* and maize, root systems grew significantly more in response to nitrogen and phosphorus starved conditions respectively using split pot experiments (Ruffel et al., 2011; Wang et al., 2020). Additionally, *PHO1* has been identified in GWAS for RSA traits in *A. thaliana* (Rosas et al., 2013), and *AMT5* has been identified in maize for striga resistance, which is likely root-based (Adewale et al., 2020). Future studies should utilize functional genetics to validate the effect of these candidate genes on maize RPF through both characterization of mutants and evaluation of natural alleles of the loci we identified in this study using near-isogenic lines.

Although *AMT5*, *SLAH2*, and *PHO1;2A* were not identified as candidate genes for RSA traits other than RPF *via* GWA, our *post hoc* analyses with the alternate SNP alleles at these genes identified significant differences in other traits such as root area, depth, and width that were consistent with their differences in RPF (Figures 7, 9, 10). This suggests that despite the independent variation among the RSA traits we measured, RPF exhibits a strong enough association with other RSA traits to identify candidate genes that significantly affect their variation as well. This connection between RPF and other RSA traits potentially helps to ameliorate the difficulty of studying the genetic basis of RSA in maize. RPF provides a relatively high throughput method for quantifying RSA and discovering candidate genes that affect multiple RSA traits in mature, field grown plants.

## Conclusion

Understanding the phenotypic plasticity and genetic control that shapes RSA in maize remains a formidable challenge. This is because of the difficulty of obtaining root phenotype data across development for mature field grown plants. To evaluate the phenotypic plasticity and genetic control of RSA in maize, we used a maize diversity panel coupled with environments of contrasting irrigation levels as well as publicly available genotype matrices from next generation sequencing data. Our results implicate that: (1) root phenotypic plasticity is predictable for some maize genotypes, (2) RSA in maize is a highly complex trait controlled by many functionally diverse genes, and (3) RPF is an efficient phenotype capable of identifying candidate genes associated with variation in additional root architectural traits. Future studies using functional genetic techniques such as mutant screens and QTL mapping in RIL populations are needed to validate the candidate genes identified in this study and accurately quantify effect sizes of alleles across developmental stages and environments. Additionally, there is a need to develop field and analysis designs that improve our ability directly test for QTLxE in statistically robust genome-wide analysis. Overall, the results discussed here extend our knowledge of root phenotypic plasticity in maize at the whole-plant, and gene levels and further elucidate the highly complex genetic architecture controlling variation in maize RSA.

## Data Availability Statement

The datasets presented in this study can be found in online repositories. The names of the repository/repositories and accession number(s) can be found in the article/Supplementary Material.

## Author Contributions

PW helped acquire phenotype data, performed the GWAS, and wrote the manuscript. KL helped acquire phenotype data, performed the GxE analyses, and wrote the manuscript. KH helped acquire phenotype data and assisted with manuscript writing. JLM helped with experimental design, acquiring phenotype data, analyses, and manuscript writing. JKM designed and supervised the experiment, provided guidance for phenotypic analyses, and assisted with manuscript writing. All authors contributed to the article and approved the submitted version.

## Conflict of Interest

The authors declare that the research was conducted in the absence of any commercial or financial relationships that could be construed as a potential conflict of interest.

## Publisher’s Note

All claims expressed in this article are solely those of the authors and do not necessarily represent those of their affiliated organizations, or those of the publisher, the editors and the reviewers. Any product that may be evaluated in this article, or claim that may be made by its manufacturer, is not guaranteed or endorsed by the publisher.

## Footnotes

1
https://github.com/braverock/PerformanceAnalytics
