# Supplementary material for: Root Pulling Force Across Drought in Maize Reveals Genotype by Environment Interactions and Candidate Genes
Source: Front Plant Sci. 2022 Apr 15;13:883209. doi: 10.3389/fpls.2022.883209 (PMC9051544; doi:10.3389/fpls.2022.883209)
Supplement: Supplementary file 1 [file Data_Sheet_1.PDF]

Supplementary Figure S1. Q-Q plot assessing p-value distributions for the GWA conducted on the fully irrigated post-flowering RPF measurement. Blue squares indicate the comparison of the expected -log10(p) (X axis) values versus the observed -log10(p) (y axis) values. The red line indicates the null hypothesis of complete concordance between expected and observed -log10(p) values.

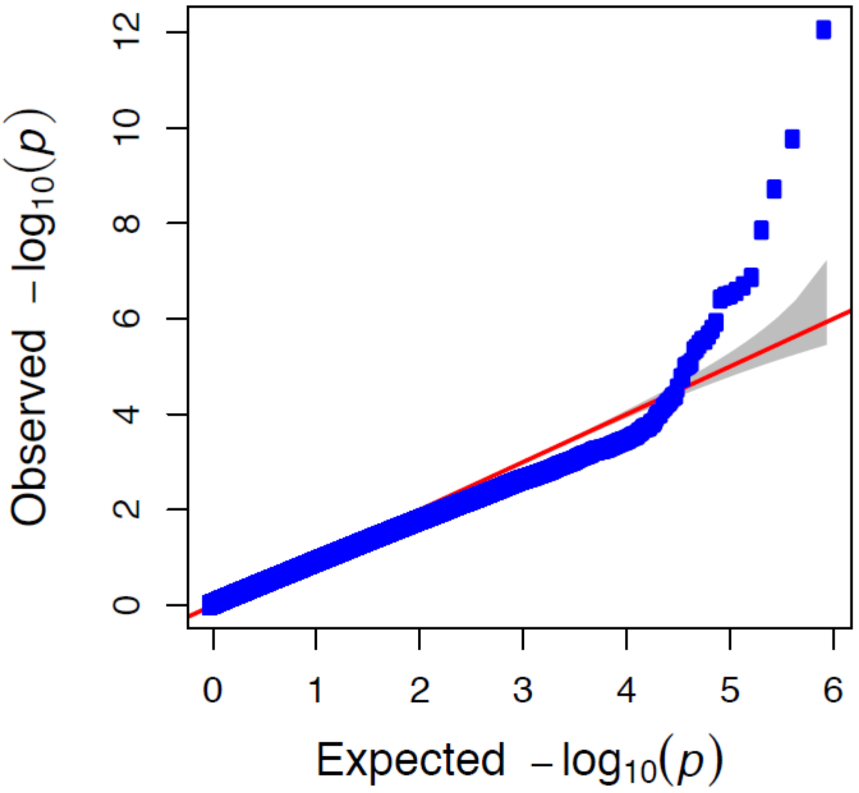

Supplementary Figure S2. Comparison of RSA for pulled and shovel excavated root systems. A, Crown root mass for the two extraction methods (mean ± SE, *n* = 42-44; P = 0.84, paired t test). B, Image-based root system architecture traits extracted by the different methods for three genotypes with varying root form (left, sample images; right, average values for root area, depth and width (mean ± SE, n = 2)). For all three of the traits, there was a significant effect of genotype (ANOVA, P < 0.01) but not of extraction method (P > 0.10).

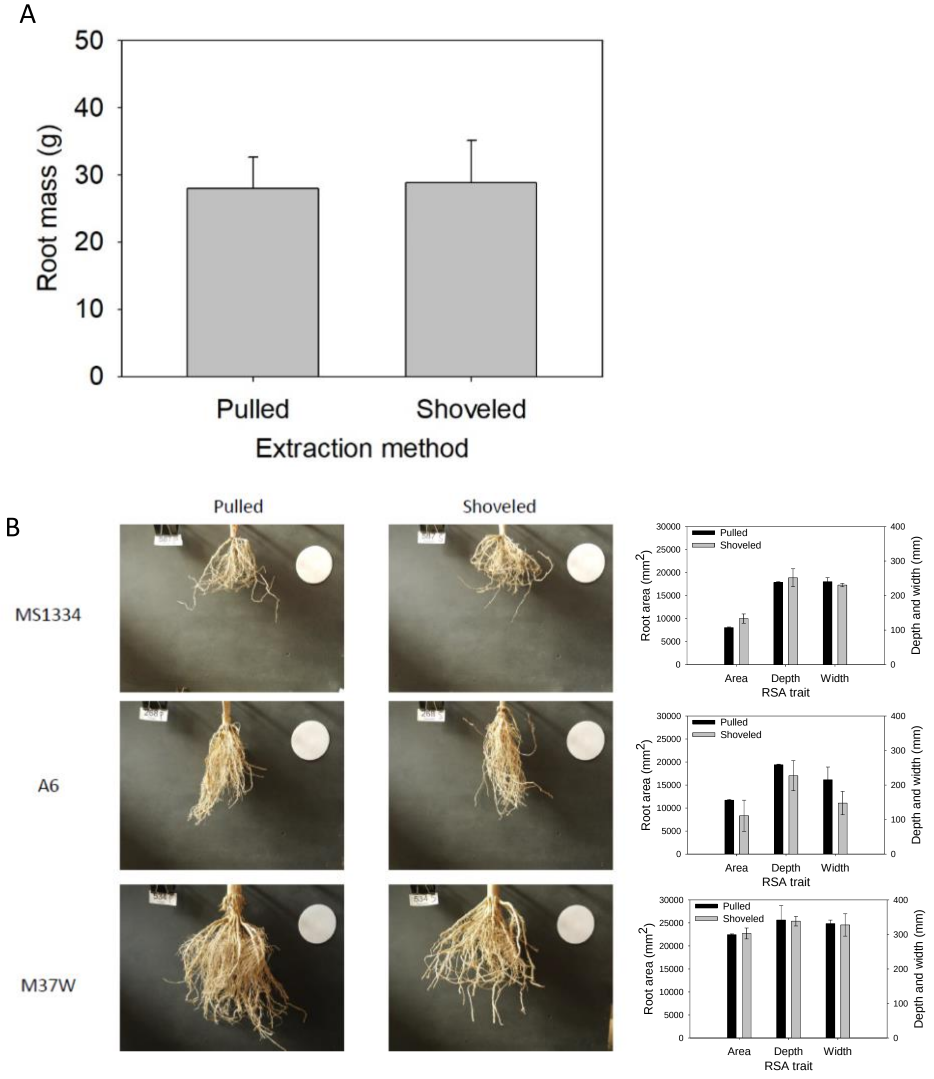

Supplementary Figure S3. Variance component analysis of traits from the 2018 and 2019 field seasons.

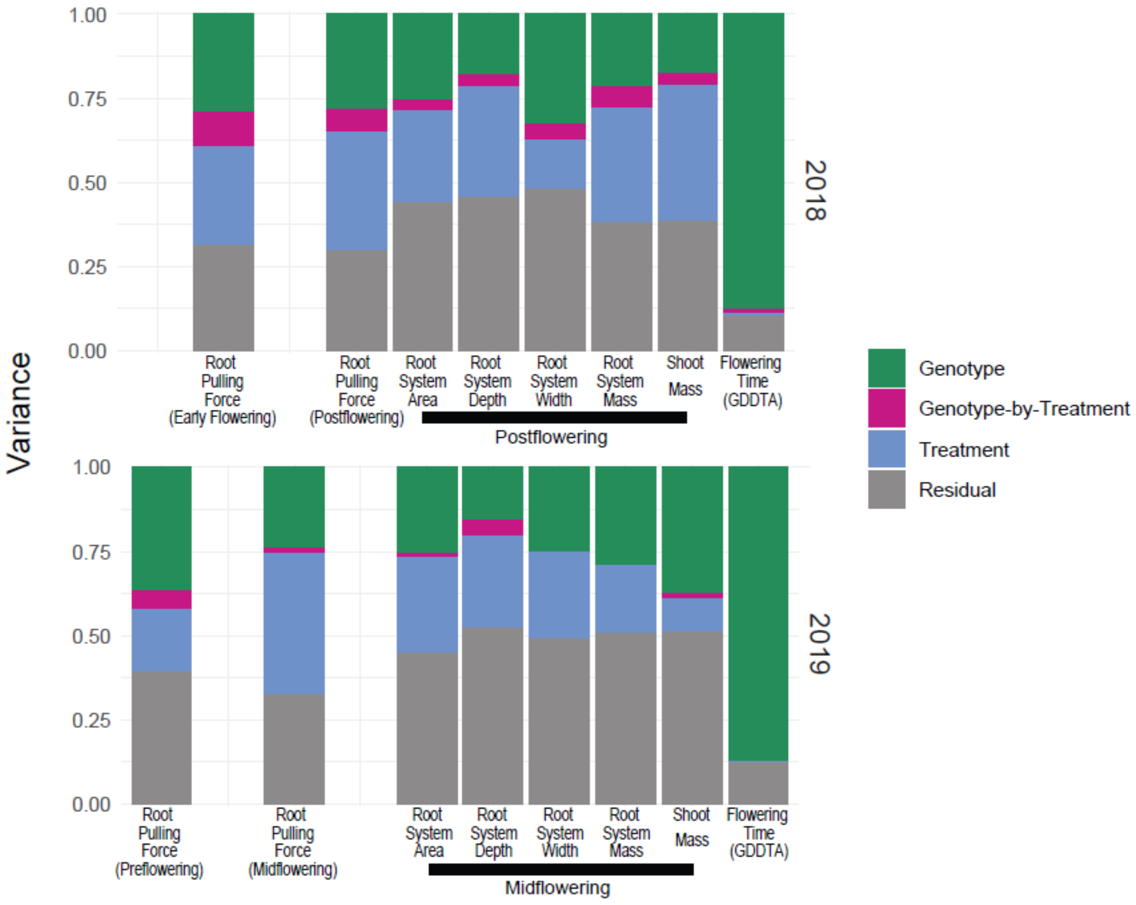

Supplementary Table S1. Summary of weather and irrigation for the four environments. FI, full irrigation; LI, limited irrigation.

Environment	June			July			August		
	Ave Temp (°C)	Precip (mm)	Irrigation (mm)	Ave Temp (°C)	Precip (mm)	Irrigation (mm)	Ave Temp (°C)	Precip (mm)	Irrigation (mm)
2018 FI	20.9	21	104	22.0	38	133	20.4	3	140
2018 LI	20.9	21	76	22.0	38	44	20.4	3	0
2019 FI	17.7	75	51	22.4	33	114	21.8	14	102
2019 LI	17.7	75	25	22.4	33	25	21.8	14	51

Supplementary Table S2. Genotypic correlations for the 2018 field season. Values are Spearman's correlation coefficients.

		Full-Irrigation								Limited-Irrigation							
		RPF (Early-flowering)	RPF (Postflowering)	Root System Area (Postflowering)	Root System Depth (Postflowering)	Root System Width (Postflowering)	Root System Mass (Postflowering)	Shoot Mass (Postflowering)	Flowering Time (GDDTA)	RPF (Early-flowering)	RPF (Postflowering)	Root System Area (Postflowering	Root System Depth (Postflowering)	Root System Width (Postflowering	Root System Mass (Postflowering)	Shoot Mass (Postflowering)	Flowering Time (GDDTA)
Full Irrigation	RPF (Early-flowering)	1															
	(Postflowering)	0.6	1														
	Root System Area (Postflowering)	0.44	0.67	1													
	Depth (Postflowering)	0.46	0.51	0.68	1												
	Width (Postflowering)	0.27	0.4	0.7	0.4	1											
	Root System Mass (Postflowering)	0.39	0.59	0.63	0.56	0.33	1										
	Shoot Mass (Postflowering)	0.27	0.53	0.53	0.35	0.34	0.61	1									
	Flowering Time (GDDTA)	-0.23	0.21	0.1 (NS)	0 (NS)	-0.03 (NS)	0.26	0.17 (**)	1								
Limited Irrigation	RPF (Early-flowering)	0.6	0.5	0.44	0.44	0.27	0.41	0.26	-0.09 (NS)	1							
	(Postflowering)	0.52	0.63	0.49	0.43	0.33	0.48	0.33	0.23	0.59	1						
	Root System Area (Postflowering)	0.47	0.47	0.54	0.4	0.48	0.4	0.34	0.07 (NS)	0.47	0.69	1					
	Depth (Postflowering)	0.37	0.33	0.35	0.41	0.23	0.34	0.27	0.02 (NS)	0.37	0.53	0.7	1				
	Width (Postflowering)	0.32	0.34	0.42	0.26	0.53	0.27	0.27	0.05 (NS)	0.31	0.48	0.78	0.48	1			
	Root System Mass (Postflowering)	0.43	0.38	0.4	0.36	0.26	0.52	0.38	0.07 (NS)	0.5	0.65	0.72	0.52	0.56	1		
	Shoot Mass (Postflowering)	0.26	0.32	0.34	0.23	0.29	0.41	0.54	0.12 (*)	0.31	0.51	0.57	0.4	0.47	0.64	1	
	Flowering Time (GDDTA)	-0.2	0.23	0.11 (NS)	-0.03 (NS)	-0.01 (NS)	0.28	0.21	0.96	-0.1 (NS)	0.23	0.09 (NS)	0.03 (NS)	0.08 (NS)	0.08 (NS)	0.13 (*)	1
	All others are (***)																

Supplementary Table S3. Genotypic correlations for the 2019 field season. Values are Spearman's correlation coefficients.

		Full-Irrigation								Limited-Irrigation							
		RPF (Preflowering)	RPF (Midflowering)	Root System Area (Midflowering	Root System Depth (Midflowering	Root System Width (Midflowering)	Root System Mass (Midflowering)	Shoot Mass (Midflowering)	Flowering Time (GDDTA)	RPF (Preflowering)	RPF (Midflowering)	Root System Area (Midflowering)	Root System Depth (Midflowering)	Root System Width (Midflowering)	Root System Mass (Midflowering	Shoot Mass (Midflowering)	Flowering Time (GDDTA)
Full Irrigation	RPF (Preflowering)	*															
	RPF (Midflowering)	0.54	*														
	Area (Midflowering)	0.49	0.76	*													
	Root System Depth (Midflowering)	0.39	0.58	0.76	*												
	Root System Width (Midflowering)	0.32	0.57	0.76	0.5	*											
	Root System Mass (Midflowering)	0.46	0.73	0.74	0.56	0.51	*										
	Shoot Mass (Midflowering)	0.49	0.72	0.63	0.48	0.45	0.7	*									
	Flowering Time (GDDTA)	-0.39	-0.05 (NS)	-0.15 (**)	-0.22	-0.07 (NS)	-0.08 (NS)	-0.2	*								
Limited Irrigation	RPF (Preflowering)	0.64	0.52	0.52	0.47	0.36	0.48	0.51	-0.36	*							
	RPF (Midflowering)	0.49	0.63	0.5	0.39	0.41	0.5	0.43	0.05 (NS)	0.5	*						
	Area (Midflowering)	0.44	0.58	0.56	0.36	0.47	0.56	0.47	-0.05 (NS)	0.49	0.75	*					
	Root System Depth (Midflowering)	0.35	0.42	0.4	0.38	0.32	0.43	0.3	0.03 (NS)	0.41	0.64	0.75	*				
	Root System Width (Midflowering)	0.3	0.47	0.47	0.26	0.55	0.41	0.35	0.04 (NS)	0.37	0.57	0.76	0.52	*			
	Root System Mass (Midflowering)	0.45	0.55	0.52	0.44	0.39	0.61	0.47	0.01 (NS)	0.48	0.73	0.77	0.66	0.53	*		
	Shoot Mass (Midflowering)	0.44	0.47	0.39	0.31	0.35	0.44	0.58	-0.11 (**)	0.45	0.63	0.6	0.46	0.46	0.69	*	
	Flowering Time (GDDTA)	-0.36	-0.05 (NS)	-0.15 (**)	-0.24	-0.06 (NS)	-0.06 (NS)	-0.2	0.94	-0.34	0.04 (NS)	-0.05 (NS)	0.04 (NS)	0.04 (NS)	0 (NS)	-0.12 (**)	*
	All others are (***)																

Supplementary Table S4. List of significant SNPs for root traits.

Trait	Marker_SNP_v2	Chromosome	B73_v5_Position	P.value	FDR_Adjusted_P.value	MAF	SNPxTreatment_pvalue	Closest_Gene	NCBI_ID	Annotation	Other_Genes_in_Interval
Area_2019_Midflowering_Limited_Irrigation	1_28862553	1	28800386	1.11E-08	0.00238393	0.244514	0.668875	Zm00001eb009320	LOC103633057	wall-associated_receptor_kinase-like_14	
RPF_2018_Earlyflowering_Full_Irrigation	1_52633486	1	52761549	2.27E-07	0.019535448	0.193215	0.53757	Zm00001eb015420	LOC100283111	Mitochondrial_import_receptor_subunit_TOM40-1	
RPF_2018_Postflowering_Full_Irrigation	1_70850891	1	71369452	1.40E-08	0.003013183	0.113772	0.2327565	NA	NA	NA	NA
Area_2019_Midflowering_Limited_Irrigation	1_71607329	1	72169664	3.47E-07	0.033217763	0.34326	0.89038	Zm00001eb019800	LOC100274688	pco121831_E3_ubiquitin-protein_ligase_BRE1-like_2	NA
Area_2018_Postflowering_Full_Irrigation	1_88530587	1	89661777	1.68E-12	1.45E-06	0.332308	0.02402	Zm00001eb023020	LOC100274725	NADH_dehydrogenase_ubiquinone_1_beta_subcom	NA
Area_2019_Midflowering_Limited_Irrigation	1_174644987	1	178766465	1.76E-12	1.52E-06	0.268025	0.441026	Zm00001eb031970	LOC100192777	bsu1-brassinosteroid_insensitive_suppressor_protein	NA
RPF_2019_Midflowering_Limited_Irrigation	1_184632834	1	188819897	5.03E-08	0.01444064	0.277429	0.6597556	NA	NA	NA	NA
RPF_2018_Earlyflowering_Full_Irrigation	1_195779532	1	200108340	9.08E-12	3.91E-06	0.160767	0.3411	Zm00001eb037030	LOC100279791	uncharacterized	NA
Root_Mass_2018_Postflower_Full_Irrigation	1_228254933	1	233758700	7.35E-10	0.000316679	0.072131	0.0001781	Zm00001eb044840	NA	npi447a_similar_to_Arabidopsis_alpha-galactosidase	NA
RPF_2018_Earlyflowering_Limited_Irrigation	1_240090714	1	245781661	1.00E-07	0.012325527	0.141813	0.4664	Zm00001eb047830	LOC103643560	protein_NETWORKED_1D	NA
RPF_2018_Earlyflowering_Full_Irrigation	1_257144174	1	263703479	4.34E-07	0.031157166	0.479351	0.0111868	Zm00001eb052050	LOC100277915	uncharacterized	NA
Area_2018_Postflowering_Full_Irrigation	1_284011654	1	290807894	3.62E-07	0.044557601	0.066154	0.05264	Zm00001eb059630	LOC100282761	inositolphosphorylceramide-B_C-26_hydroxylase	Zm00001eb059620
RPF_2019_Midflowering_Limited_Irrigation	1_286452650	1	293268580	5.98E-09	0.002576697	0.084639	0.973803	Zm00001eb060250	LOC542228	ao1-aldehyde_oxidase1	NA
RPF_2018_Postflowering_Full_Irrigation	2_5214470	2	5439373	3.14E-07	0.032160857	0.426647	0.2872388	Zm00001eb068370	LOC103645845	UDP_glycosyltransferase_89B1_like	Zm00001eb068380
RPF_2018_Earlyflowering_Full_Irrigation	2_189571970	2	194795692	1.39E-12	1.20E-06	0.10767	0.1304	Zm00001eb101840	NA	znf13_zinc_finger_protein13	Zm00001eb101820
Area_2018_Postflowering_Full_Irrigation	2_191968855	2	197925572	3.81E-08	0.008373172	0.183077	0.25848	Zm00001eb102380	LOC100282453	uncharacterized	NA
RPF_2018_Postflowering_Limited_Irrigation	2_194905510	2	201300073	1.12E-10	4.83E-05	0.44883	0.857392	Zm00001eb103300	LOC100216563	Ypt_Rab_GAP_domain_of_gyp1p_superfamily_protei	NA
RPF_2018_Postflowering_Limited_Irrigation	2_225678072	2	232124026	9.73E-09	0.002795563	0.093567	0.447575	Zm00001eb114070	LOC100193577	bsdtf1_BSD_transcription_factor_1	Zm00001eb114080
Area_2018_Postflowering_Full_Irrigation	3_2764530	3	2752924	8.30E-10	0.000357746	0.449231	0.2643875	Zm00001eb119560	LOC103649556	probable_inactive_receptor_kinase_At4g23740	Zm00001eb119570_Zm00001eb119580
RPF_2018_Postflowering_Limited_Irrigation	3_36837474	3	37036459	6.68E-11	4.83E-05	0.070175	0.6562497	Zm00001eb127840	LOC100281498	c3h12_C3H_transcription_factor_312	NA
RPF_2018_Earlyflowering_Limited_Irrigation	3_124189168	3	125626205	6.09E-08	0.012325527	0.488304	0.9587	Zm00001eb136170	LOC100273335	LOC100193821_like_pseudogene	Zm00001eb136160
Area_2019_Midflowering_Limited_Irrigation	3_148281418	3	150367677	2.30E-07	0.024747692	0.26489	0.212652	Zm00001eb140740	LOC100279357	CRR4	NA
Width_2018_Postflower_Limited_Irrigation	3_197373364	3	202551404	1.65E-09	0.000711957	0.417647	0.97851	Zm00001eb153060	LOC100191195	uncharacterized	NA
RPF_2018_Postflowering_Full_Irrigation	3_213390029	3	218974411	1.36E-07	0.023492807	0.275449	0.184741	Zm00001eb158100	LOC100193891	umi8_ustilago_maydis_induced8	NA
RPF_2018_Postflowering_Limited_Irrigation	3_216464843	3	221942005	8.35E-08	0.011996347	0.061404	0.6772497	Zm00001eb159080	LOC100216899	50S_ribosomal_protein_L31_chloroplastic_like	NA
RPF_2018_Earlyflowering_Full_Irrigation	3_217665275	3	223191889	1.23E-09	0.000264767	0.287611	0.0195096	Zm00001eb159490	LOC103651474	S_type_anion_channel_SLAH2	Zm00001eb159500
Area_2019_Midflowering_Limited_Irrigation	4_3484831	4	4464213	1.86E-11	8.03E-06	0.257053	0.6195707	Zm00001eb165770	LOC100284245	lipase	NA
RPF_2018_Earlyflowering_Full_Irrigation	4_4737624	4	5770814	6.93E-11	1.99E-05	0.154867	0.34157	Zm00001eb166390	LOC100193315	uncharacterized	NA
Shoot_Mass_2018_Postflower_Full_Irrigation	4_5240306	4	6257841	1.37E-11	1.18E-05	0.321543	0.04413	Zm00001eb166700	LOC100272411	Monosaccharide_sensing_protein_2	Zm00001eb166690
RPF_2019_Midflowering_Full_Irrigation	4_5240306	4	6257841	2.05E-07	0.019592705	0.322785	0.06776	Zm00001eb166700	LOC100272411	Monosaccharide_sensing_protein_2	Zm00001eb166690
Area_2019_Midflowering_Limited_Irrigation	4_14856169	4	16195853	1.33E-07	0.020531198	0.18652	0.6834557	Zm00001eb168910	LOC606409	pdi1_protein_disulfide_isomerase1	NA
RPF_2019_Midflowering_Full_Irrigation	4_15603122	4	16930219	3.54E-08	0.005394407	0.310127	0.556292	Zm00001eb169030	LOC103652889	putative_disease_resistance_protein_At1g50180	NA
Width_2018_Postflower_Limited_Irrigation	4_44023834	4	46160868	6.83E-08	0.014709043	0.247059	0.69795	NA	NA	NA	NA
RPF_2018_Postflowering_Full_Irrigation	4_147900944	4	153130434	3.36E-07	0.032160857	0.067365	0.552648	Zm00001eb185930	LOC100282580	ATP_dependent_RNA_helicase_dhh1	NA
RPF_2018_Earlyflowering_Full_Irrigation	4_157505381	4	162758639	5.26E-08	0.006478282	0.352507	0.6608009	Zm00001eb187730	LOC100276787	platz7_PLATZ_transcription_factor_7	NA
Width_2018_Postflower_Limited_Irrigation	4_165834025	4	170913660	9.38E-08	0.016164092	0.173529	0.770226	Zm00001eb189900	LOC100383369	hb115_Homeobox_transcription_factor_115	NA
Area_2018_Postflowering_Full_Irrigation	4_167193841	4	172282498	3.48E-07	0.044557601	0.069231	0.0867912	Zm00001eb190390	LOC100501623	uncharacterized	Zm00001eb190380
RPF_2018_Postflowering_Full_Irrigation	4_171921792	4	177077448	1.71E-10	7.37E-05	0.073353	0.0616928	Zm00001eb191650	LOC103654129	phos2_phosphate_transporter2	Zm00001eb191660_Zm00001eb191670
RPF_2018_Earlyflowering_Full_Irrigation	4_180673390	4	185608069	3.65E-07	0.028593682	0.199115	0.3396186	Zm00001eb194170	LOC103654247	TPR_repeat_containing_thioredoxin_TTL4	NA
Shoot_Mass_2018_Postflower_Full_Irrigation	4_186948136	4	192358302	3.02E-08	0.008669373	0.207395	0.003268	Zm00001eb196380	LOC103654328	probable_calcium_transporting_ATPase_8_plasma_m	Zm00001eb196390
Root_Mass_2018_Postflower_LimitedIrrigation	4_187603761	4	193002656	3.10E-10	0.00026712	0.291667	0.71712	Zm00001eb196590	LOC100281546	AMP_binding_protein	NA
Width_2018_Postflower_Limited_Irrigation	4_193341102	4	199504085	7.08E-09	0.002032925	0.302941	0.92018	Zm00001eb198220	LOC100275453	uncharacterized	Zm00001eb198230_Zm00001eb198240
Root_Mass_2018_Postflower_LimitedIrrigation	4_196928694	4	203027401	1.16E-08	0.004988518	0.447531	0.50234	Zm00001eb199080	LOC103654457	WEB_family_protein_chloroplastic_like	Zm00001eb199070
RPF_2018_Earlyflowering_Limited_Irrigation	4_238999665	4	247718490	9.50E-08	0.012325527	0.055556	0.255737	Zm00001eb208840	LOC100191624	RuvB_like_protein_1	m00001eb208850_Zm00001eb208860_Zm00001eb20883
RPF_2018_Earlyflowering_Full_Irrigation	4_239477872	4	248172395	9.02E-09	0.001553863	0.137168	0.3115	Zm00001eb209190	LOC100501509	xat7_xylan_alpha_1_3_arabinofuranosyl_transferase	Zm00001eb209180_Zm00001eb209200
RPF_2018_Earlyflowering_Full_Irrigation	5_5029498	5	5336509	9.19E-07	0.049934413	0.433628	0.1600393	Zm00001eb213510	LOC113218479	uncharacterized	Zm00001eb213500_Zm00001eb213490
RPF_2018_Postflowering_Full_Irrigation	5_18699967	5	19629132	2.04E-07	0.029301865	0.332335	0.0410501	Zm00001eb219240	LOC103626058	uncharacterized	Zm00001eb219250
Root_Mass_2018_Postflower_Full_Irrigation	5_51435988	5	53136167	1.74E-10	0.000150013	0.22459	0.238844	Zm00001eb226000	LOC100274986	uncharacterized	Zm00001eb226010
RPF_2019_Midflowering_Full_Irrigation	5_61227311	5	63063966	8.07E-11	6.96E-05	0.071203	0.0112587	Zm00001eb228060	LOC100282640	protein_BRE	Zm00001eb228070
RPF_2019_Midflowering_Full_Irrigation	5_66112461	5	68090202	7.22E-08	0.008888191	0.09019	0.262433	Zm00001eb229190	LOC100127010	a2_anthocyaninless2	NA
Root_Mass_2018_Postflower_LimitedIrrigation	5_151631304	5	154800023	7.02E-08	0.02017197	0.195988	0.75382	Zm00001eb239530	LOC100280577	rps24_ribosomal_protein_S24	NA
RPF_2018_Earlyflowering_Limited_Irrigation	5_171562659	5	174940230	4.23E-09	0.003644646	0.346491	0.203737	Zm00001eb243430	NA	NA	NA
RPF_2018_Postflowering_Limited_Irrigation	5_199375500	5	207318113	2.10E-07	0.025807257	0.229532	0.4804843	Zm00001eb251400	LOC100191236	uncharacterized	Zm00001eb251410
RPF_2019_Midflowering_Full_Irrigation	5_204088246	5	212134701	2.58E-08	0.005394407	0.148734	0.6816638	Zm00001eb253130	LOC100273249	uncharacterized	Zm00001eb253140
RPF_2018_Postflowering_Full_Irrigation	5_211939338	5	220320408	3.88E-07	0.03345518	0.088323	0.4012506	Zm00001eb256890	LOC100276172	uncharacterized	Zm00001eb256900
RPF_2019_Midflowering_Full_Irrigation	6_88908042	6	98631379	9.16E-08	0.009861784	0.254747	0.0170504	Zm00001eb273440	LOC541654	mir2_maize_insect_resistance2	Zm00001eb273450
RPF_2018_Postflowering_Full_Irrigation	6_100125636	6	110333672	1.93E-09	0.000553035	0.136228	0.00293	Zm00001eb276250	LOC100383495	uncharacterized	NA
RPF_2018_Earlyflowering_Full_Irrigation	6_150686807	6	161845903	4.81E-07	0.031448089	0.29351	0.26961	Zm00001eb289310	LOC100274162	uncharacterized	Zm00001eb289300_Zm00001eb289320
RPF_2018_Earlyflowering_Full_Irrigation	6_156523621	6	167982260	1.21E-07	0.013079861	0.056047	0.0979338	Zm00001eb291820	NA	NA	Zm00001eb291810
RPF_2018_Earlyflowering_Full_Irrigation	7_5087423	7	5253849	5.11E-07	0.031448089	0.123894	0.188734	Zm00001eb299950	LOC103631941	WPP_domain_associated_protein	NA
RPF_2018_Postflowering_Limited_Irrigation	7_137339567	7	141950448	1.74E-08	0.003743774	0.160819	0.7180342	Zm00001eb317490	LOC103632986	uncharacterized	NA
Area_2019_Midflowering_Limited_Irrigation	7_143397759	7	149546828	2.23E-07	0.024747692	0.070533	0.8931777	Zm00001eb319700	LOC100280671	uncharacterized	NA
RPF_2018_Postflowering_Limited_Irrigation	7_147349220	7	153752462	4.85E-08	0.008357043	0.067251	0.7754057	NA		NA	NA
Shoot_Mass_2018_Postflower_Full_Irrigation	8_14794756	8	15239785	9.91E-10	0.000426911	0.117363	0.0007953	Zm00001eb335970	LOC103634830	glutathione_hydrolase_3	NA
Width_2018_Postflower_Limited_Irrigation	8_27634189	8	28400131	2.76E-10	0.00023756	0.445588	0.592249	Zm00001eb339070	LOC101027215	reticulon_like_protein_B1	Zm00001eb339080
RPF_2019_Midflowering_Full_Irrigation	8_130850637	8	134449811	3.67E-08	0.005394407	0.101266	0.212141	Zm00001eb354880	LOC541960	lg4_liguleless4	NA
RPF_2018_Earlyflowering_Limited_Irrigation	8_151660348	8	155586037	2.27E-08	0.009787305	0.111111	0.2971861	Zm00001eb360570	LOC100273686	DUF1639_family_protein	Zm00001eb360560
RPF_2018_Earlyflowering_Full_Irrigation	8_170775256	8	177271533	3.51E-08	0.005048028	0.109145	0.72921	Zm00001eb369000	LOC103636557	protein_LURP_one_related_8	Zm00001eb369010
RPF_2019_Midflowering_Full_Irrigation	9_20476910	9	22326943	3.76E-08	0.005394407	0.343354	0.3213958	Zm00001eb377330	LOC100192617	LOC103639077_like_pseudogene	NA
RPF_2018_Earlyflowering_Limited_Irrigation	9_20746005	9	22603129	8.71E-08	0.012325527	0.353801	0.780701	Zm00001eb377430	LOC100283048	hydrolase_NUDIX_family_protein	Zm00001eb377440_Zm00001eb377420
Area_2018_Postflowering_Full_Irrigation	9_89811890	9	95147061	3.89E-08	0.008373172	0.089231	0.17492	Zm00001eb386320	LOC103638686	uncharacterized	Zm00001eb386310
Area_2019_Midflowering_Limited_Irrigation	9_93448924	9	98782362	8.70E-09	0.00238393	0.191223	0.1029268	Zm00001eb386920	LOC100273774	Isoleucine_tRNA_ligase_cytoplasmic	Zm00001eb386910
RPF_2018_Earlyflowering_Full_Irrigation	9_125169873	9	130889232	1.49E-07	0.014227845	0.439528	0.79248	Zm00001eb393630	LOC100281071	uncharacterized	NA
Area_2019_Midflowering_Limited_Irrigation	9_126197406	9	131931139	1.43E-07	0.020531198	0.286834	0.24829	Zm00001eb393900	LOC100284249	pc326_plasmacytoma_326_homolog	NA
RPF_2019_Midflowering_Limited_Irrigation	9_145770205	9	151987481	7.33E-12	6.32E-06	0.166144	0.8183319	Zm00001eb399800	LOC100193428	hsftf9_HSF_transcription_factor_9	Zm00001eb399780
RPF_2018_Earlyflowering_Limited_Irrigation	9_147849857	9	154222038	1.35E-07	0.014498242	0.233918	0.2231	Zm00001eb400730	LOC103639462	syntaxin_61	NA
RPF_2018_Earlyflowering_Full_Irrigation	10_9225000	10	9052337	9.27E-07	0.049934413	0.297935	0.4014349	Zm00001eb407780	NA	oy1_oil_yellow1	NA
RPF_2019_Midflowering_Full_Irrigation	10_10730275	10	10598470	3.75E-09	0.001617289	0.131329	0.3158832	Zm00001eb408280	LOC100193740	apx11_ascorbate_peroxidase11	NA
Root_Mass_2018_Postflower_Full_Irrigation	10_82028112	10	83192826	1.96E-08	0.005619811	0.295082	0.39104	Zm00001eb416420	LOC100279207	putative_RING_zinc_finger_domain_superfamily_pro	NA
RPF_2018_Postflowering_Full_Irrigation	10_132025153	10	134586330	8.79E-13	7.58E-07	0.182635	0.03674	Zm00001eb427000	LOC100381734	amt5_ammonium_transporter5	NA
RPF_2018_Postflowering_Full_Irrigation	10_135053540	10	137674814	2.68E-07	0.032160857	0.142216	0.210005	Zm00001eb428170	LOC100273313	flz35_FCS_like_zinc_finger35	NA
Area_2018_Postflowering_Full_Irrigation	10_135059844	10	137681118	3.50E-07	0.044557601	0.049231	0.19472	Zm00001eb428170	LOC100273313	flz35_FCS_like_zinc_finger35	NA
RPF_2018_Earlyflowering_Limited_Irrigation	10_145178896	10	147333905	3.85E-07	0.03683975	0.204678	0.6562809	Zm00001eb432010	LOC100194321	Coatomer_subunit_epsilon_2	Zm00001eb432020
RPF_2018_Earlyflowering_Limited_Irrigation	10_146188124	10	148418125	6.36E-08	0.012325527	0.080409	0.4899	Zm00001eb432510	NA	mha4_proton_exporting_ATPase4	Zm00001eb432500

Supplementary Table S5. List of significant SNPs for flowering time.

Trait	Marker_SNP_v2	Chromosome	B73_v5_Position	P.value	FDR_Adjusted_P.value	SNPxTreatment_pvalue
GDDTA_2018_Full_Irrigation	1_22384346	1	22570469	3.86E-08	0.006653651	0.99931
GDDTA_2018_Limited_Irrigation	1_34595396	1	34525723	4.82E-10	8.31E-05	0.709035
GDDTA_2018_Full_Irrigation	1_34595396	1	34525723	7.91E-08	0.00852531	0.709035
GDDTA_2018_Full_Irrigation	1_234059072	1	239779155	7.90E-07	0.044034128	9.39E-01
GDDTA_2018_Full_Irrigation	1_234059181	1	239779264	5.18E-07	0.034357417	0.639911
GDDTA_2018_Full_Irrigation	1_234122303	1	239841401	7.50E-07	0.044034128	0.855298
GDDTA_2018_Full_Irrigation	1_234122368	1	239841466	8.18E-07	0.044034128	0.869005
GDDTA_2018_Full_Irrigation	1_244469214	1	250149814	4.95E-08	0.007106611	0.86884
GDDTA_2019_Full_Irrigation	1_244469214	1	250149814	7.60E-08	0.007398732	0.92121
GDDTA_2018_Full_Irrigation	1_272275573	1	278827623	1.67E-12	1.43E-06	0.802781
GDDTA_2018_Full_Irrigation	2_37419164	2	38969540	1.36E-07	0.012322113	0.985472
GDDTA_2018_Full_Irrigation	2_45967533	2	47778943	2.10E-07	0.016421007	0.75916
GDDTA_2018_Limited_Irrigation	2_152921504	2	157561349	3.45E-07	0.029717962	0.515189
GDDTA_2018_Full_Irrigation	2_152921504	2	157561349	1.43E-07	0.012322113	0.515189
GDDTA_2019_Full_Irrigation	2_194166189	2	200268685	5.45E-08	0.007398732	0.7564203
GDDTA_2018_Limited_Irrigation	3_18009555	3	18026393	2.77E-09	0.00039751	0.56572
GDDTA_2019_Full_Irrigation	3_176805743	3	181618571	3.71E-08	0.007398732	0.8431
GDDTA_2018_Full_Irrigation	3_202829071	3	208156072	6.59E-10	0.000283747	0.91432
GDDTA_2018_Limited_Irrigation	3_210522634	3	216109871	3.21E-11	1.39E-05	0.33557
GDDTA_2018_Limited_Irrigation	3_222671752	3	228285300	3.26E-09	0.00040083	0.2432276
GDDTA_2018_Full_Irrigation	3_222671752	3	228285300	1.45E-09	0.000415397	0.2432276
GDDTA_2019_Full_Irrigation	3_222671752	3	228285300	5.14E-08	0.007398732	0.9276
GDDTA_2019_Full_Irrigation	4_22329446	4	24042781	2.47E-07	0.017754335	0.86708
GDDTA_2019_Full_Irrigation	4_136058402	4	141143400	8.59E-08	0.007398732	0.89482
GDDTA_2018_Limited_Irrigation	4_142201504	4	147390958	2.02E-07	0.01930074	0.6881
GDDTA_2018_Limited_Irrigation	5_2811674	5	3128652	6.16E-07	0.048262206	0.6314
GDDTA_2019_Full_Irrigation	5_8454996	5	8970718	1.21E-08	0.003477176	0.4914411
GDDTA_2019_Limited_Irrigation	5_16851434	5	17615597	3.62E-10	0.000311745	0.5392
GDDTA_2019_Limited_Irrigation	5_54920199	5	56670866	1.82E-09	0.000410152	0.861649
GDDTA_2018_Limited_Irrigation	5_92354630	5	94465080	5.16E-11	1.48E-05	0.837323
GDDTA_2018_Limited_Irrigation	5_199138822	5	207087752	2.07E-12	1.78E-06	0.3566
GDDTA_2019_Limited_Irrigation	6_94761834	6	104558680	1.33E-09	0.000410152	0.9458
GDDTA_2019_Full_Irrigation	6_142661184	6	153795816	8.42E-08	0.007398732	0.99969
GDDTA_2019_Limited_Irrigation	6_148970518	6	160088684	2.84E-08	0.004888121	0.844
GDDTA_2019_Limited_Irrigation	6_153260064	6	164451679	5.49E-08	0.007883181	0.582
GDDTA_2018_Full_Irrigation	7_137162241	7	142051397	6.78E-08	0.008343274	0.70898
GDDTA_2019_Full_Irrigation	7_152441704	7	161442936	2.43E-07	0.017754335	0.9345
GDDTA_2018_Full_Irrigation	8_131779375	8	135384583	2.11E-09	0.000454472	0.675565
GDDTA_2018_Limited_Irrigation	8_132047205	8	135657401	3.79E-08	0.004077727	0.46263
GDDTA_2018_Full_Irrigation	8_161713363	8	168032736	5.05E-07	0.034357417	0.50173
GDDTA_2019_Full_Irrigation	8_161713363	8	168032736	7.86E-08	0.007398732	0.75648
GDDTA_2019_Full_Irrigation	9_50389650	9	54220507	1.08E-09	0.000463887	0.9994
GDDTA_2019_Full_Irrigation	9_121718186	9	127302804	3.29E-07	0.02179468	0.97423
GDDTA_2019_Full_Irrigation	9_144317637	9	150543792	5.69E-10	0.000463887	0.6514
GDDTA_2018_Limited_Irrigation	9_151977644	9	158369935	3.79E-10	8.17E-05	0.874404
GDDTA_2019_Limited_Irrigation	9_154893380	9	161266976	1.24E-07	0.015269731	0.542036
GDDTA_2019_Limited_Irrigation	10_14912510	10	15010034	1.90E-09	0.000410152	0.9725
GDDTA_2019_Full_Irrigation	10_14912510	10	15010034	4.07E-07	0.025041165	0.9725
